# Characterization of doxycycline-mediated inhibition of Marfan syndrome-associated aortic dilation by multiphoton microscopy

**DOI:** 10.1038/s41598-020-64071-8

**Published:** 2020-04-28

**Authors:** Arash Y. Tehrani, Jason Z. Cui, T. Bucky Jones, Ester Hotova, Monica Castro, Pascal Bernatchez, Cornelis van Breemen, Mitra Esfandiarei

**Affiliations:** 1 Centre for Heart Lung Innovation, University of British Columbia, Vancouver, BC V6Z 1Y6 Canada; 2 Department of Anesthesiology, Pharmacology & Therapeutics, University of British Columbia, Vancouver, BC V6T 1Z3 Canada; 3 Department of Cardiothoracic Surgery, School of Medicine, Stanford University, Palo Alto, CA 94304 USA; 4 Department of Anatomy, College of Graduate Studies, Midwestern University, Glendale, AZ 85308 USA; 5 Department of Biomedical Sciences, College of Graduate Studies, Midwestern University, Glendale, AZ 85308 USA

**Keywords:** Animal disease models, Cardiovascular models, Skin models, Biological fluorescence, Cardiovascular diseases, Cardiovascular biology, Biological techniques, Biophysics, Physiology, Structural biology, Diseases, Cardiovascular diseases

## Abstract

Marfan syndrome (MFS) is a connective tissue disorder that results in aortic root widening and aneurysm if unmanaged. We have previously reported doxycycline, a nonselective matrix metalloproteinases (MMPs) inhibitor, to attenuate aortic root widening and improve aortic contractility and elasticity in MFS mice. We were also first to use multiphoton microscopy, a non-invasive and label-free imaging technique, to quantify and link the aortic ultrastructure to possible changes in the skin dermis. Here, we aimed to assess the effects of long-term doxycycline treatment on the aortic ultrastructure and skin dermis of MFS mice through immunohistochemical evaluation and quantification of elastic and collagen content and morphology using multiphoton microscopy. Our results demonstrate a rescue of aortic elastic fiber fragmentation and disorganization accompanied by a decrease in MMP-2 and MMP-9 expression within the aortic wall in doxycycline-treated MFS mice. At 12 months of age, reduced skin dermal thickness was observed in both MFS and control mice, but only dermal thinning in MFS mice was rescued by doxycycline treatment. MMP-2 and MMP-9 expression was reduced in the skin of doxycycline-treated MFS mice. A decrease in dermal thickness was found to be positively associated with increased aortic root elastin disorganization and wall thickness. Our findings confirm the beneficial effects of doxycycline on ultrastructural properties of aortic root as well as on skin elasticity and structural integrity in MFS mice.

## Introduction

Marfan Syndrome (MFS), an autosomal dominant genetic disorder affecting the connective tissues, is caused by mutations in the gene encoding the extracellular matrix (ECM) glycoprotein fibrillin-1 (FBN1)^1^. With an estimated incidence of 1 in 3,000–5,000 individuals, MFS patients exhibit prominent cardiovascular, skeletal, ocular and pulmonary abnormalities^2^. FBN1 protein monomers associate with one another to form microfibrils with structural and regulatory functions in the ECM and act as important scaffolds for elastin fiber and collagen deposition, and thus, provide structural integrity^3^. It is suggested that downstream detrimental effects of FBN1 protein abnormality in MFS is mainly due to disruption of its regulatory role of sequestering the latent form of transforming growth factor beta (TGF- β) in the extracellular space of connective tissue throughout the body^2^. In the cardiovascular system, degeneration of microfibrils lead**s** to loss of elastin fiber integrity within the blood vessel wall, resulting in aortic elastin fiber fragmentation, disorganization, and reduced load bearing capacity, all of which contribute to aortic root aneurysm, dissections, and rupture as the leading cause of mortality in patients if left untreated^4^.

Using a well-established mouse model of MFS that carries FBN-1 mutation similar to one found in human MFS patients with aortic aneurysm, our group has previously demonstrated that aortic aneurysm progression in MFS is associated with an upregulation of the matrix-degrading enzymes matrix metalloproteinase (MMP)-2 and -9, elastic fiber degeneration, compromised aortic contractility, and endothelial dysfunction^5,6^, and that long term treatment with a sub-antibiotic dose of doxycycline, a general and nonspecific inhibitor of MMPs, was effective at reducing MMPs expression levels and improving aortic wall functional and structural parameters, while blocking the progression of aortic aneurysm in a well-established mouse model of MFS^7,8^. Furthermore, we have recently demonstrated that the beneficial effects of doxycycline on MFS aortic vessels are associated with significant improvements in pulse-wave velocity (PWV), which is a reliable index of aortic wall stiffness, and aortic wall elasticity *in vivo*^9^. However, the long-term effects of doxycycline treatment on aortic elastin and collagen ultrastructure have not yet been fully elucidated. We have previously used multiphoton microscopy (MPM), an advanced label-free imaging technique, to quantify aortic fiber fragmentation and disorganization in the thoracic aorta, as well as the volumetric density of cutaneous elastin in the skin of wild type and MFS mice^10^, highlighting the notion that structural changes in the aortic wall seemed to be associated with structural changes in the skin dermis of affected mice^10^. As a follow up study, the present report aimed to use MPM to determine the long-term effects of a low sub-antibiotic dose of doxycycline on aortic elastin and collagen ultrastructure and additionally examine elastic and collagen alterations in the skin dermis of the well-established mouse model of MFS.

## Materials and methods

### Experimental animals and treatment

For animal experiments, we used a heterozygous transgenic mouse model harbouring the *Fbn1* allele mutation (*Fbn1*^*C1041G/+*^, encoding a cysteine to glycine substitution Cys^1041^ → Gly), the most common class of mutations in MFS^5,7,8,10^. Heterozygous *Fbn1*^*C1041G/+*^ mice recapitulate all aspects of phenotype observed in MFS human patients, including curvature of the spine, long bone overgrowth, elastic fiber fragmentation within the aortic wall, and progression of aortic root enlargement. Heterozygous *Fbn1*^*C1041G/+*^ mice were bred with C57BL/6 wild-type mice to generate Marfan (MFS*, Fbn1*^*C1041/+*^) and littermate control (CTRL*, Fbn1*^*+/+*^) mice, which were housed in the institutional animal facility with standard animal room conditions (25 °C, 12-hour light-dark cycle, ≤5 animals in a cage). All animal procedures were approved by the University of British Columbia animal ethics board [reference number A15–0275], and all animals received humane care in compliance with the Guide for the Care and Use of Laboratory Animals. (www.nap.edu/catalog/5140.html). The mouse model of MFS shows signs of aortic elastin fiber fragmentation and an increase in aortic pulse wave velocity (a functional index of aortic wall stiffness) around 3 months of age, which is comparable with 20-year old human MFS patient. In human MFS patients carrying the same fibrillin-1 mutations, the average age for aortic dissection/rupture is around 50-60 years of age that is comparable to 12–15 months of age in mice. Since our study aimed to look at the long-term treatment of doxycycline in MFS adults who are at a higher risk for aortic dissection or rupture, we aimed to run a long-term experiment in mice, and therefore sacrifice mice at 12 months of age. Hence, Beginning at 5–6 weeks of age, mice (n = 4–5) were randomly assigned to CTRL ± doxycycline hyclate (Alfa Aesar, Ward Hill, MA; 0.24 g/L/day in drinking water) and MFS ± doxycycline hyclate groups for the duration of the study as described previously^7,9^.

Due to doxycycline instability and sensitivity to light, the doxycycline water was shielded from light and changed every other day throughout the length of treatment. Mice were sacrificed at 12 months of age under inhaled terminal anesthesia (3.5% v/v isoflurane at 1.5 L O_2_) followed by cervical dislocation, and aortic and skin tissues were collected for various experimental assays.

### Tissue preparation for histologic and multiphoton imaging

Thoracic aortas were dissected out from experimental mice at 12 months of age and washed in cold Phosphate Buffered Saline (PBS, pH 7.4). A single transverse section (1–2 mm) was cut from the ascending aortic root above the level of the aortic valve for use in MPM. The transverse aortic root sections were placed vertically on a petri dish and immersed in PBS for MPM imaging. Some aortic sections (2 mm) were embedded in paraffin blocks and sectioned at 5μm thickness for immunohistochemical staining. Furthermore, the skin from the dorsal surface was also dissected out from the same experimental subjects after hairs were removed by very gentle application of hair removal lotion, followed by washing the skin in cold PBS. The harvested skin was flattened out and cut into two pieces. One half was used for MPM imaging and the other was formalin fixed for immunohistochemical staining. Skin samples were also stained for hematoxylin and eosin (H&E) to assess dermal thickness.

### Features of multiphoton imaging system

**Figure 1:**
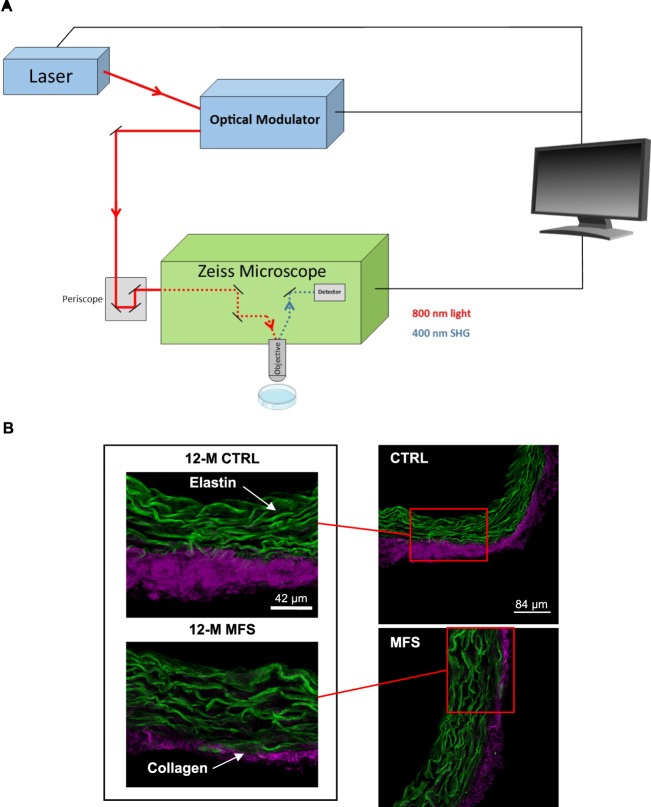
Multiphoton microscopy (MPM) technique on the aortic root of CTRL and MFS mice. (**A**) A basic schematic of the multiphoton microscopy system. A laser beam is focused on the specimen samples through a high-resolution objective to excite them. Backscatter TPF and SHG signals emitted from aortic and skin tissue are collected through the same objective and directed towards the detectors. Detectors can then differentiate the TPF and SHG signals associated with collagen and elastin respectively. **(B)** Representative MPM images of aortic root sections of CTRL and MFS mice at 12 months of age (12-M) show both TPF (green for elastin) and SHG (purple for collagen) signals.

A basic schematic of the MPM system used in this report is illustrated (Fig. 1A) and the fundamentals of a MPM system capable of both two-photon excited fluorescence (TPF) and second harmonic generation (SHG) signal detection has previously been described in detail^11^. The MPM system consisting of an inverted super resolution microscope (Carl Zeiss LSM 880) with a Ti:Sapphire infrared pulse laser (Coherent Chameleon Ultra) was used to generate two-photon excited fluorescence from elastin and collagen in the samples. With an excitation wavelength of 920 nm, SHG signals originating from collagen were obtained from the emission wavelength of 490 nm. TPF signals originating from elastin were obtained from the emission wavelength range of 400–650 nm with a peak at 500 nm as previously described^10^.

### Quantitative analysis of multiphoton images

3-D reconstruction of aortic MPM images, as well as the quantification of volumetric density of elastin and collagen signals was performed using Volocity 6.3 imaging software (PerkinElmer, Waltham, MA) as previously described^12^. Elastic fiber fragmentation was determined using Aperio ImageScope software (Leica Biosystems, Wetzlar, Germany) by tracing individual fibers and measuring their length in pixels followed by unit conversion into micrometers^13^. A two-dimensional (2-D) Fast Fourier Transform (FFT) algorithm was utilized to determine elastic fiber orientation and anisotropy. Briefly, this technique converts complex spatial patterns of intensity and position from aortic images into directional frequency components using MATLAB software (Mathworks, Natick, MA). The details of this technique along with the theory of FFT algorithm are explained in further detail in our previous work^10^.

### Immunohistochemistry

Immunohistochemical staining of aortic and skin tissues was performed with the Abcam Mouse and Rabbit Specific HRP/DAB IHC Detection Kit (cat. no. ab236466) per the manufacturer instructions. Briefly, tissue sections were deparaffinized in Histoclear (Fisher Scientific cat. no. 50–899–90147), and rehydrated through a series of descending ethanol solutions. After antigen retrieval in IHC-TEK Epitope Retrieval Solution (cat no. 1W-1100), slides were incubated at 4 °C overnight with the following primary antibodies purchased from Abcam: MMP-2 (1:200; ab38898), and MMP-9 (1:200; ab37150). After washing in 0.5 M Tris buffer, slides were incubated at room temperature for 60 minutes with biotinylated secondary antibody followed by development with DAB solution per the kit instructions. Slides were then dehydrated, cleared, and coverslipped. Negative controls included parallel processing of slides with omission of the primary antibody. Slides were imaged at 200X using a Zeiss Axiostar Microscope. The digitized images were then analyzed using Image J software (NIH). A rectangular sampling tool (~8500 μm^2^) was applied to five regularly spaced areas of the aortic ring, and the average positive signal intensity within the multiple regions of interest (ROI = 5) was obtained using the auto-threshold tool. The proportional area of stained tissue for each analyzed section was determined by averaging the five ROIs.

### Statistical analysis

Graph Pad Prism software 8.2 was used for all analyses. Values are expressed as mean ± standard error of the mean (SEM). Comparisons between two groups were made by two-tailed Student’s *t*-test, and one-way analysis of variance (ANOVA) was used to compare three or more groups followed by Tukey’s Post-hoc test to correct for multiple comparisons with a *P* < 0.05 value considered significant.

## Results

### Visualization of aortic elastic fibers and collagen with MPM

In this study, we utilized MPM (Fig. 1A) to investigate the morphology and ultrastructure of elastic fibers and collagen in the aortic root of our mice at 12 months of age. We have previously demonstrated the morphological changes that occur in elastic fiber morphology in younger CTRL and MFS mice as assessed with MPM; namely, increased elastic fiber fragmentation and disorganization observed in the aortic root of MFS mice as early as 6 months of age with progressive worsening up to 9 months of age^10^. Here, we assessed the aortic structure in aged 12-months old mice. Representative cross-sectional images of aortic roots taken with MPM are shown to demonstrate the quality of images obtained (Fig. 1B). Elastic fibers captured through TPF signal are represented in green and collagen fibers captured through SHG represented in purple (Fig. 1B).

### Assessment of volumetric density of elastin and collagen in the aortic root

**Figure 2:**
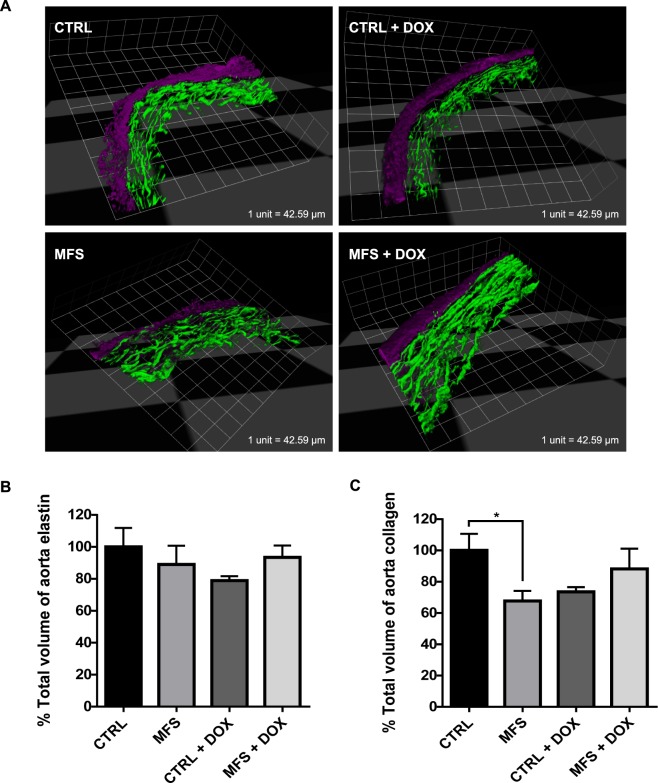
Assessment of volumetric density of elastin and collagen in the aortic root. (**A**) Representative 3-D reconstructed images of aortic root sections from CTRL and MFS mice at 12 months of age. Elastin is shown in green (TFP signal) and collagen in purple (SHG signal). Bar graphs represent quantification of volumetric density of aortic **(B)** elastin and **(C)** collagen. No significant differences from the CTRL in elastin or collagen density were found in the aortic root**s** of mice following treatment with doxycycline (DOX). Data presented as mean ± SEM; n = 4–5, *p < 0.05 is considered significant.

3-D structures of CTRL and MFS aortic roots were reconstructed from stacks of MPM images to determine the volumetric density of elastin and collagen (Fig. 2A). No significant differences were observed in the volumetric density of total elastin between 12-month old CTRL and MFS aortic root samples in the absence or presence of doxycycline treatment (Fig. 2B). MFS mice showed a decrease in total aortic collagen at 12 months of age compared to CTRL mice; however, doxycycline treatment had no effect on total collagen content (Fig. 2C). These findings are in agreement with our previous work, where no significant differences were observed between CTRL and MFS at 3, 6, or 9 months of age^10^. These findings suggest that measures of elastin volumetric densities in the aortic wall are not ideal for determining morphological differences between CTRL and MFS aorta in mice.

### Effect of doxycycline on aortic elastic fiber fragmentation, disorganization, and wall thickening

**Figure 3:**
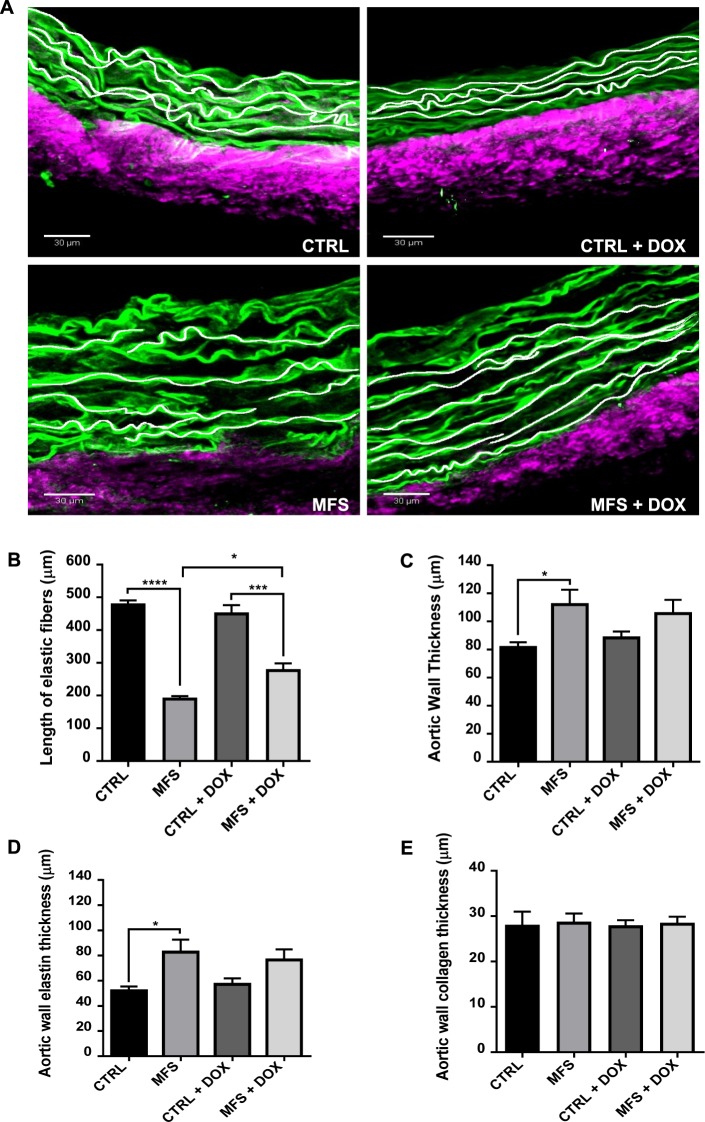
Quantitative assessment of aortic fiber fragmentation and vessel thickness. (**A**) Representative MPM images showing tracing of elastic fibers in aortic root sections from CTRL and MFS mice at 12 months of age following treatment with doxycycline (DOX). **(B)** Comparison of average elastin fiber lengths as an assessment of the degree of elastic fiber fragmentation following treatment with DOX. **(C)** Total vessel wall thickness in the aortic roots of CTRL and MFS mice. **(D,E)** The total vessel wall thickness is further stratified into its two main components of elastin and collagen thickness to determine where vessel remodeling is contributing to total vessel thickening. Data presented as mean ± SEM; n = 4–5, *p < 0.05, ***p < 0.001, ****p < 0.0001 is considered significant.

Aortic wall remodeling, including fragmentation, disorganization, and thickening of medial elastic fibers is a key histological feature of the MFS aortic root^4^. In this part of the study, individual elastic fibers were traced to determine the degree of fragmentation in the medial areas of aortic rings isolated from treated and untreated CTRL and MFS mice at 12 months of age (Fig. 3A). Our data show that at 12 months of age, untreated MFS mice exhibit approximately a 60% decrease in average elastic fiber length compared to CTRL mice, and that doxycycline treatment significantly improves elastic fiber length in MFS aortic roots by ~31% compared to non-treated MFS (Fig. 3B). Aortic medial thickening, an established hallmark of MFS aortic vessels due to excessive matrix turnover at sites of elastin loss, was measured in CTRL and MFS aorta. When left untreated, aortic wall thickness in 12-month old MFS mice shows a significant increase (around 31%) as compared to CTRL mice (Fig. 3C). The difference in wall thickness is not detectable in doxycycline-treated MFS and CTRL mice (Fig. 3C). Interestingly, the thickening of the aortic medial layer seems to be associated with an increase in elastin fiber thickness (Fig. 3D), but not with an increase in collagen deposition in the adventitial layers (Fig. 3E).

**Figure 4:**
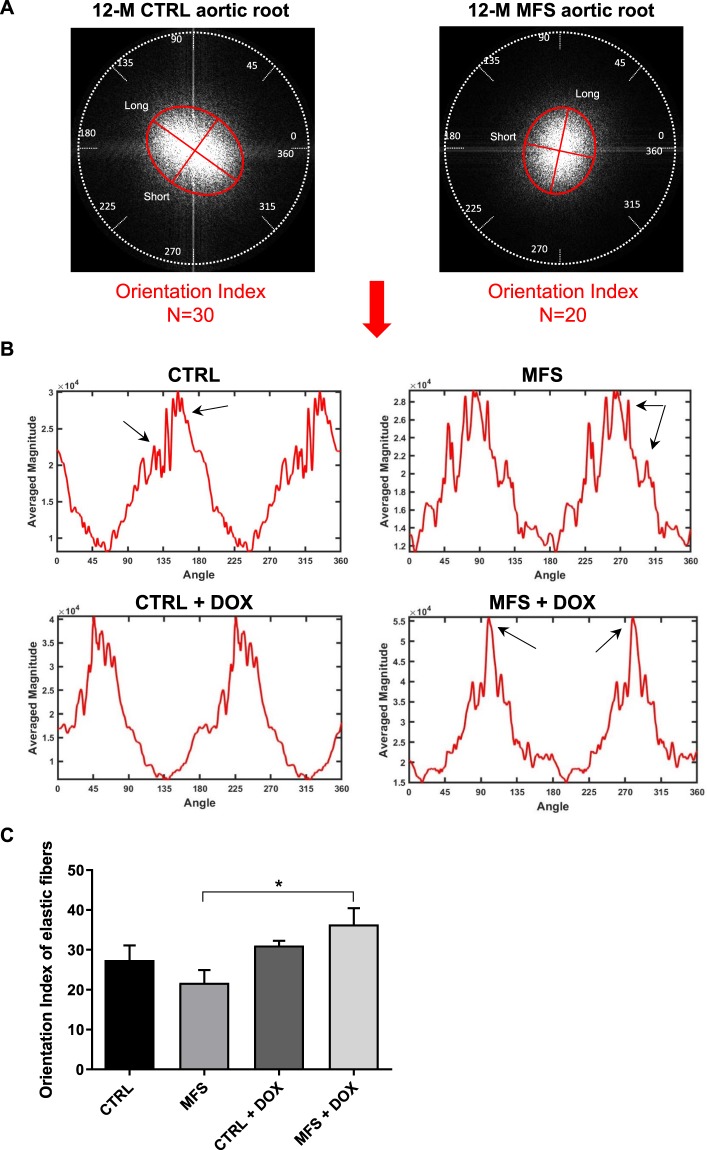
Analysis of aortic elastic fiber organization by FFT algorithm. (**A**) Representative image**s** showing the intensity signals from the aorta of CTRL and MFS mice at 12 months of age plotted in 2-D frequency spaces as long and short axes. The orientation index (N), was calculated from the ratio of the short axis to the long axis, representing the level of order. **(B)** Following the frequency space transfer, the relationship between fiber angular direction (angular values from 0 to 360 degrees transcribed from the radar grid in the 2-D frequency) and intensity signals (average frequency magnitude) was analyzed. Elastin signals appear as two major peaks on each graph with additional jagged and rough peaks revealed on the shoulders of the major peaks (double arrows) in both CTRL and MFS mice. An increase in number and spread of peaks is representative of increased elastin fiber disorganization whereas the peaks appear cleaner and more organized following doxycycline treatment. **(C)** Average orientation indices of aortic root elastic fibers in 12-month old CTRL and MFS mice with and without doxycycline (DOX) treatment. Data presented as mean ± SEM; n = 4–5, *p < 0.05 is considered significant.

We next looked at the degree of elastic fiber disorganization in the aortic root isolated from 12-month old MFS and CTRL mice using an FFT algorithm. To determine individual fiber directions, the spatial frequencies of each fiber were first plotted in a 2-D frequency space on a long and short axis (Fig. 4A). For comparison between groups, the ratio of the short axis to the long axis was calculated to yield a fiber orientation index. The orientation index ranges from 0 to 100, representing the levels of order; the higher the number, the greater the degree of organization (Fig. 4A). As shown by the representative images (Fig. 4A), the orientation index in the aorta of a 12-month old CTRL mouse (N = 30) is more than the one in MFS counterpart (N = 20). Furthermore, the 2-D analysis shows the relationship between the elastin fiber’s angular direction and magnitude from representative CTRL and MFS mice from each group (Fig. 4B). The x-axis represents all possible angular positions of individual elastin fibers on a radial scale between 0–360 degrees plotted against the average magnitude or the number of fibers on the y-axis. The angular signal intensity of untreated CTRL and MFS aortic roots display major peaks (Fig. 4B; black arrows) spread out at a multitude of angles, with maximum average magnitudes of 2–3 ×10^4^ equating to elastic fibers being oriented in many directions representative of a higher level of disorganization. This effect was reversed following treatment with doxycycline as both the shift in angular direction and the average magnitudes of MFS aortas are increased with less noise in the peaks (Fig. 4B; black arrows). This represents the reversal of fiber disorganization following doxycycline treatment with the majority of elastic fibers being now oriented bi-directionally representative of a higher level of fiber organization. There are slight variations in the orientation of elastic fibers as multiple images are acquired at different z-planes from the same aortic root. Thus, the average orientation index (N) for each aortic segment was determined from multiple z-stack images. Quantification of these results shows doxycycline treatment effectively increased the N value for fiber orientation index in MFS aortas bringing it to a level that is not different from CTRL aorta (Fig. 4C).

### Effect of doxycycline on skin dermal thickness

**Figure 5:**
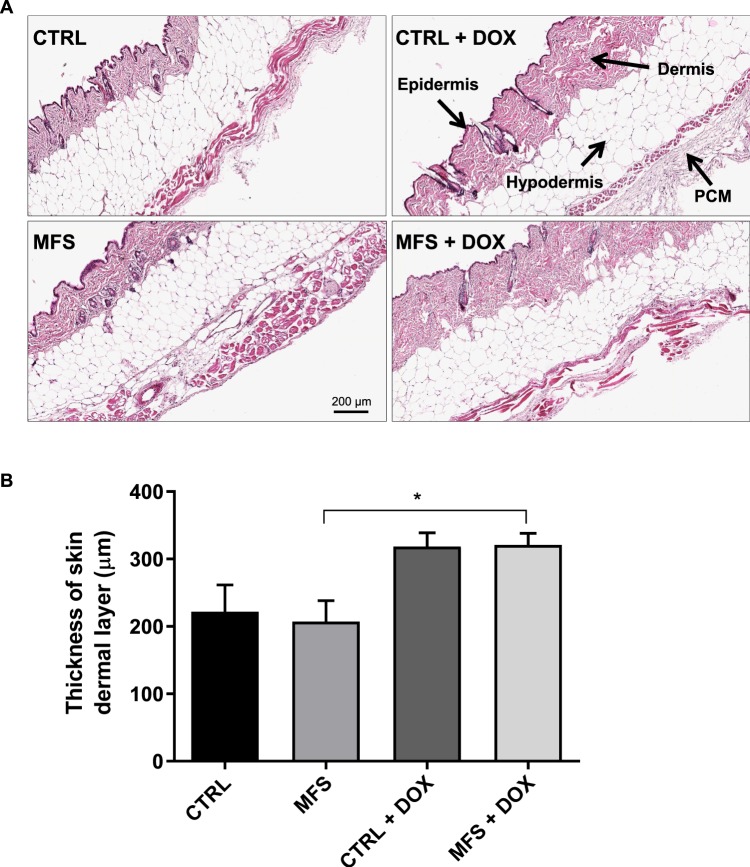
Quantitative assessment of skin dermal thickness. (**A**) Representative H&E staining of skin from CTRL and MFS mice at 12 months of age following treatment with or without doxycycline (DOX). Basic layering of the mouse skin includes the epidermis, dermis, hypodermis, and panniculus carnosus muscle (PCM). **(B)** Measurement of skin dermal thickness of CTRL and MFS mice. Data presented as mean ± SEM; n = 4–5, *p < 0.05 is considered significant.

Sections of skin from CTRL and MFS mice were stained with H&E to observe changes in skin morphology that might be taking place in the dermis (Fig. 5A). The dermal layer of 12-month old CTRL and MFS mice show relatively equal levels of thickness, likely due to dermal thinning of CTRL mice as a result of natural aging. Interestingly, treatment with doxycycline significantly prevents age-associated dermal thinning in MFS mice (Fig. 5B). Although not reaching significance, a similar effect on dermis thickness was also observed in 12-month old CTRL mice treated with doxycycline.

### Effects of doxycycline on MMP expression in aortic wall and skin

**Figure 6:**
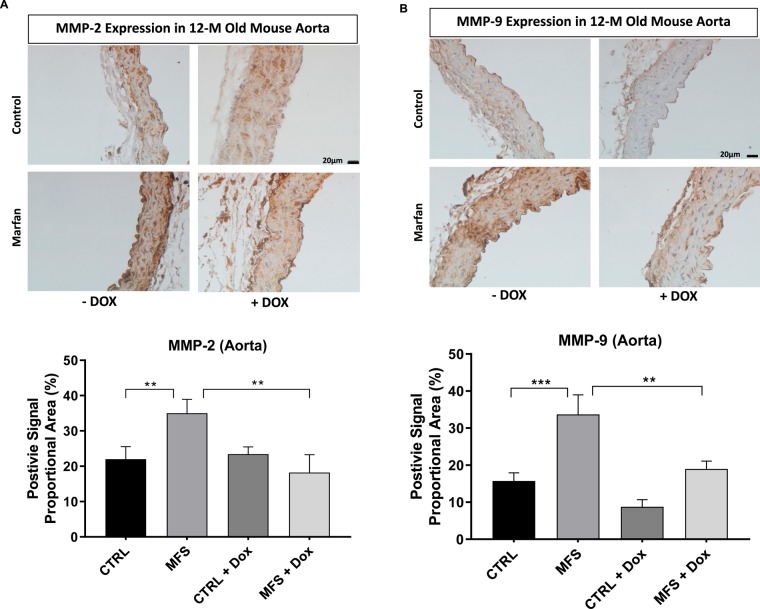
Effect of doxycycline (DOX) treatment on the expression of MMP-2 and MMP-9 in the aorta. Immunohistochemistry staining images and bar graphs representing the expression of **(A)** MMP-2 and **(B)** MMP-9 in the aortic roots of CTRL and MFS mice at 12 weeks of age treated with or without DOX. Data presented as mean ± SEM; n = 5, **p  <  0.01, ***p  <  0.001 is considered significant.

**Figure 7:**
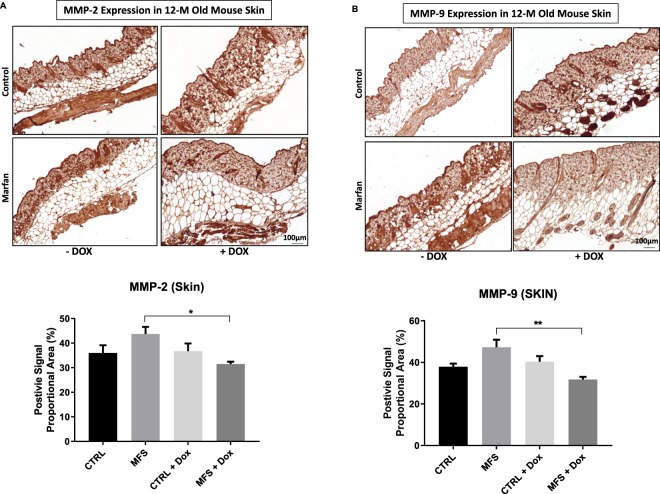
Effect of doxycycline (DOX) treatment on the expression of MMP-2 and MMP-9 in the skin. (**A**) The upper panel shows representative MMP-2 staining of skin from CTRL and MFS mice at 12 months of age with or without DOX, and the lower panel shows bar graphs representing the expression of MMP-2 in the skin of CTRL and MFS mice at 12 weeks of age with or without DOX. Data presented as mean ± SEM; n = 5, *p < 0.05 is considered significant. **(B)** The upper panel shows representative MMP-9 staining of skin from CTRL and MFS mice at 12 months of age with or without DOX treatment. The lower panel shows bar graphs representing the expression of MMP-9 in the skin of CTRL and MFS mice at 12 weeks of age treated with or without DOX. Data presented as mean ± SEM; n = 5, **p  <  0.01 is considered significant.

Immunohistochemistry was performed on aortic sections from experimental animals to detect the expression of MMP-2 and MMP-9. In agreement with previous work, aortic sections from MFS mice show a significant increase in the expression of MMP-2 (Fig. 6A) and MMP-9 (Fig. 6B) as compared to CTLR aortas, and treatment with doxycycline reduces the expression of MMP-2 and MMP-9 to levels observed in CTRL samples (Fig. 6A,B). Given the loss of elastin in the skin along with the progressive dermal thinning observed in both aging CTRL and MFS mice, we measured the expression of MMPs in the skin (Fig. 7A,B) in order to determine if doxycycline exerted similar effects to those observed in the aorta. We found no significant differences in the expression levels of MMP-2 (Fig. 7A) and MMP-9 (Fig. 7B) in the dermal skin of CTRL and MFS mice at 12 months of age. Interestingly, doxycycline treatment significantly reduced the expression levels for MMP-2 (Fig. 7A) and MMP-9 (Fig. 7B) in MFS dermal skin, but the same phenomenon was not observed in CTRL mice.

### Correlation between aortic and skin pathology in MFS mice

**Figure 8:**
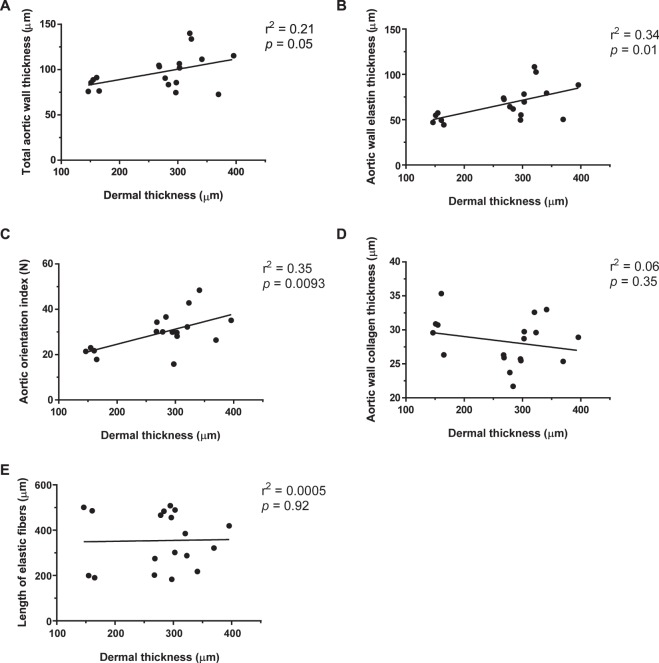
Correlation plots of aortic parameters versus dermal thickness. The dermal thicknesses of mice are plotted against a number of different aortic parameters assessed in this study to determine possible correlations between the two. From the aortic parameters that were compared to dermal thickness, only **(A)** total aortic wall thickness, **(B)** aortic wall elastin thickness, and **(C)** aortic orientation index were found to be significantly correlated with dermal thickness. However, the thickness of **(D)** aortic wall collagen thickness and **(E)** aortic elastic fiber length were not associated, and did not show any positive correlation with dermal thickness. Each point on the plot represents one mouse, *p < 0.05 is considered significant.

The dermal thickness of the experimental mice was compared to a number of aortic parameters to establish possible links between the progression of aortic pathology and changes in skin dermal thickness (Fig. 8). The parameters of interest include total wall aortic thickness, aortic wall elastin thickness, aortic wall collagen thickness, aortic orientation index, and length of aortic elastin fibers. From the aortic parameters that were compared to dermal thickness, total aortic wall thickness (Fig. 8A), aortic wall elastin thickness (Fig. 8B), and aortic orientation index (Fig. 8C) were all found to be significantly correlated to dermal thickness; where an increase in skin dermal thickness was positively correlated with an increase in aortic wall and orientation index. Interestingly, wall collagen thickness (Fig. 8D) and aortic elastic fiber length (Fig. 8E) were not associated with dermal thickness.

## Discussion

Cardiovascular complications, specifically thoracic aortic aneurysm and dissection, represent the majority of morbidity and mortality factors in MFS^14^. Although the exact molecular pathophysiology of MFS-associated aortic widening is not completely understood, the progressive nature of the disease is generally accredited to the structural abnormality of FBN-1 microfibrils leading to perturbation of the TGF-β signaling cascade and the upregulation of downstream MMPs which contribute to medial degeneration observed in MFS^15^. The vascular abnormalities associated with MFS have been well characterized in both MFS patients and mouse models. These abnormalities include fragmentation and disorganization of elastic fibers, aortic medial thickening due to increased deposition of ECM components, increased aortic stiffness as measured by PWV, and endothelial dysfunction^4,5,16,17^. MFS management guidelines have long recommended the use of anti-hypertensive β-blockers and/or angiotensin II (Ang II) receptor type 1 (ATR1) blockers (ARBs) for reducing hemodynamic stress against the aortic wall and in the case of ARBs, to also indirectly downregulate excessive TGF-β signaling^18^. Unfortunately, clinical data available on β-blockers and ARBs in MFS are scarce and inconsistent, with recent systematic and meta-analyses deeming no benefit of these medications in MFS patients^19,20^. However very recently, the AIMS trial has shown an association with a reduction in the rate of aortic dilation in MFS following treatment with the ARB irbesartan^21^. Despite a reduction in aortic root diameter rates, there is still an apparent overall increase in aortic root diameter over time. Thus, due to the lack of effective available management options, there is a need for more effective alternative management therapies in MFS as either mono- or concomitant therapies.

Doxycycline, a nonspecific broad spectrum MMP inhibitor, has been shown by our group and others to be more effective than the current “gold standard” β-blocker atenolol at attenuating MFS-associated aortic widening, and improving elastic fiber integrity and vascular function in the MFS mouse through its inhibition of MMP-2 and -9^7,22,23^. More recently, we have shown that long-term doxycycline treatment could also correct PWV starting after 18 weeks of treatment, a clinically relevant determinant of changes in aortic wall stiffness and elasticity, which is closely associated with disease progression^9^. Although previous reports have characterized the anti-remodelling effects of doxycycline treatment on elastic fiber integrity in the aorta of MFS mice, these data were analysed using classical histological techniques involving tissue fixation and staining which can alter native vessel morphology^5,7,8,23^. This is particularly important in the case of MFS where elastic fiber integrity is already compromised, and where structural analysis mainly involves quantification of the degree of elastic fiber fragmentation or breaks. Furthermore, these studies grade elastic fiber fragmentation and disorganization based on semi-quantitative scoring systems that introduce a wide degree of human error and inter-observer variation. Our group was the first to characterize both elastin and collagen contents in the aorta of MFS mice using MPM^10^. As a follow up study to our previous report, in the present study, we sought to quantitatively assess the anti-remodelling effects of long-term doxycycline treatment on the aortic ultrastructure of an established MFS mouse model using MPM^10^. This will compliment our recently reported comprehensive *in vivo* evaluation of aortic function and structure in live MFS mice subjected to a long-term treatment with doxycycline using ultrasound imaging^9^.

MPM is a novel and elegant imaging technique that provides several advantages over classical histological methods including specificity, sensitivity, and no requirements for fixation and rough processing of delicate tissues. This is possible due to the nature of elastin and collagen being naturally occurring fluorophores, which can be differentiated by their generation of SHG and TPF signals, respectively^24,25^. In this study, we assessed and determined ultrastructural parameters of the aorta including elastic fiber fragmentation, medial thickening, and total volumetric density of elastin and collagen. Our current data in 12-month old mice showed no significant differences in measurements of total volume of elastin in the aortic root. This is in agreement with our previous observations, where no differences were observed in volumetric densities of elastin in aortic samples from younger (3-, 6-, and 9-month old) CTRL and MFS mice, suggesting this to be an unsatisfactory parameter for assessing the changes associated with the progression of aortic aneurysm in MFS mice^10^, and thus, an ineffective measure for assessing the potential beneficial effects of doxycycline treatment. It is important to note that the normal elasticity of the aortic root, which needs to withstand the mechanical stress of pulsatile blood flow, is determined predominantly by the structural integrity of elastic fibers found in the media of arteries, where fragmentation and disorganization of these elastic fibers can lead to dysfunctional blood vessels. Therefore, in this study we have focused on assessing the integrity of elastic fibers in the aortic root using MPM.

We have previously shown that elastic fiber fragmentation in the aortic root of MFS mice begins to accelerate at 6 months of age compared to healthy CTRL subjects^10^. In this study, using the calculated average length of aortic elastin fibers as a surrogate of elastic fragmentation, we found elastic fibers to be heavily fragmented in the aortic root of aged MFS mice compared to CTRL mice at 12 months of age. Elastin fragmentation was significantly attenuated in MFS mice treated with doxycycline. Interestingly, doxycycline did not influence the degree of aortic medial thickening commonly observed in MFS, suggesting an inherent limitation in efficacy of doxycycline’s anti-remodelling properties in MFS.

Since the orientation and organization of elastic fibers can determine important functional properties of blood vessels, including resistance to strain and load bearing strength^26,27^, we decided to assess the degree of aortic elastic fiber disorganization using the FFT algorithm as previously described^10^. In our previous report we showed that at 3, 6, and 9 months of age, the orientation indices (indications of fiber disorganization) for elastin fibers, were significantly reduced as compared to healthy counterparts^10^. There was also a trend of decreasing orientation index of CTRL aging mice from 3 to 9 months of age^10^. Our present data shows that at 12 months of age, CTRL and MFS aorta fail to show significant differences in orientation index with approximate values of N = 20–25 for both groups. This lack of difference in the orientation index between CTRL and MFS groups at 12 months of age can be explained by postulating that MFS exhibits accelerated aging processes, which eventually converges with normal aging. Treatment with doxycycline significantly attenuated the decrease in orientation index and elastic disorganization in MFS mice. Interestingly, doxycycline did not exert the same effect on rescuing elastic organization of aging but otherwise healthy CTRL vessel, which may suggest other mechanisms to be involved in breakdown of elastic fibers in natural aging.

We have previously reported that MFS mice show thinning of skin due to a loss of elastin as early as 3 months of age^10^. One of the hallmarks of aging involves the decrease of skin thickness, an organ that contains the highest level of elastin and collagen expression in the body. Given doxycycline’s beneficial effects on preserving elastic fiber integrity and organization within the aortic wall, we decided to assess the levels of skin elastin content and dermal thickness in our mice to determine whether inhibition of MMPs expression and activity would have any effect. However, we were unable to detect differences in total elastin content between untreated MFS and CTRL skin samples by MPM, likely because of a high degree of elastin breakdown due to both normal and accelerated aging, given that we have previously shown that at 9 months of age, both CTRL and MFS mice lose a significant amount of their skin elastin content^10^. As we reported before, the decline in dermal thickness in CTRL mice starts at around 3 months of age (~450 μm) and is dramatically decreased at 9 months of age (~200 μm), which is comparable to levels observed in age- and sex-matched MFS counterparts^10^. In this study, we show the dermal thickness of both CTRL and MFS groups to be ~200 μm and equal to each other at 12 months of age,. Doxycycline treatment significantly attenuated dermal thinning in MFS-mice. Although not reaching significance, a similar effect on dermis thickness was also observed in 12-month old CTRL mice treated with doxycycline, re-emphasizing the notion that the mechanisms involved in MFS-associated dermal thinning may be similar in some aspects to those of natural aging. To study causation, we measured the expression of MMP-2 and MMP-9, the two MMPs, shown to be increased in the MFS aorta. Despite a trend towards increased MMP expression in untreated MFS versus CTRL skin, we did not observe any significant differences in expression of MMP-2 and MMP-9 in the dermis and epidermis of skin samples collected from untreated CTRL and MFS mice at 12 months of age. Interestingly, doxycycline treatment was able to reduce the expression levels of MMP-2 and MMP-9 in treated MFS mice skin, but not in treated CRTL subjects. This may suggest that signaling pathways other than MMPs are also involved in normal aging of skin in CTRL mice, while MMPs are playing roles that are more prominent in pre-mature and accelerated aging of the skin in MFS mice.

Given doxycycline’s robust effect on the attenuation of skin dermal thinning in MFS mice, we assessed if any correlations could be made between several aortic parameters measured in this study versus the degree of dermal thickness in our experimental animals. From the five parameters examined, an increase in total aortic wall thickness, aortic wall elastin thickness, and aortic orientation index were positively correlated with an increase in dermal thickness. However, aortic wall collagen thickness and the length of aortic elastic fibers showed no such association. These positive correlations of dermal thickness to parameters of aortic integrity raise the notion of a potential value of measuring skin dermal thickness in MFS patients as a possible accessory procedure for diagnostic/prognostic purposes. Further careful investigations in the animal models and MFS patients will determine whether the regular and minimally invasive assessment of structural changes in the skin could be of value in determining the state of progression of aortic pathology in these patients.

## Conclusion

The present study explores the effects of long-term treatment with a sub-antibiotic dose of doxycycline on aortic and dermal ultrastructure in a mouse model of MFS using MPM. We have found MPM to provide higher levels of detail, while reducing histological artifacts related to assessments of aortic elastic integrity in MFS mice. Doxycycline significantly reduces the expression of MMP-2 and MMP-9 accompanied by the attenuation of elastic fiber fragmentation and disorganization in the aorta of MFS mice. In addition, we found that skin dermal thinning observed in MFS is attenuated by doxycycline treatment, and that decreased thickness of the dermal layer is positively associated with increased levels of aortic fiber disorganization and medial thickening. Our findings open a new direction for testing the hypothesis that the degree of skin dermal thickness could possibly be considered as a maker for changes in aortic wall structure and integrity. Further clinical studies that allow for the examination of structural changes in the dermis and epidermis and establishing a potential correlation between dermal skin and aortic events in MFS patients can provide information about the diagnostic or prognostic values of such assessments in these patients.
